# Diagnostic utility of the revised Lake Louise criteria in myocarditis associated with active autoimmune rheumatic disease

**DOI:** 10.1016/j.jocmr.2025.101916

**Published:** 2025-06-02

**Authors:** Alina Hua, Blanca Domenech-Ximenos, Begona Lopez, Giovanni Sanna, Amedeo Chiribiri, Ronak Rajani, Michael Marber, David D'Cruz, Michelle Fernando, Tevfik F. Ismail

**Affiliations:** a School of Biomedical Engineering and Imaging Sciences, King’s College London, London, UK; b Cardiology Department, Guy’s & St Thomas’ NHS Foundation Trust, London, UK; c Department of Radiology, Hospital Clinic de Barcelona, Barcelona, Spain; d Rheumatology Department, Guy’s & St Thomas’ Hospital, London, UK

**Keywords:** Myocarditis, Lake Louise criteria, Parametric mapping, Cardiovascular magnetic resonance, Autoimmune rheumatic disease

## Abstract

**Background:**

Cardiovascular magnetic resonance (CMR) is the principal non-invasive imaging modality used to diagnose idiopathic/viral myocarditis. The revised Lake Louise criteria (LLC) stipulate that a diagnosis can be made in the presence of one T1-based and one T2-based criterion. While the LLC have been extensively validated in viral myocarditis, their utility for the diagnosis of myocarditis due to an active autoimmune rheumatic disease is unknown. This study sought to assess the performance of the revised LLC in patients with clinically suspected myocarditis due to active systemic autoimmune disease.

**Methods:**

Patients with clinically active autoimmune rheumatic disease, symptoms of myocarditis, and elevated troponin levels were recruited and compared with controls with autoimmune rheumatic disease but no suspicion of autoimmune myocarditis. All patients underwent CMR at 1.5T including T1 and T2 mapping.

**Results:**

Thirty-seven patients with suspected myocarditis due to an active autoimmune rheumatic disease were recruited with a median (interquartile [IQR]) troponin level of 121 ng/L (72–318 ng/L). Overall, 65% (24/37) of patients met either of the two revised LLC resulting in a sensitivity (95% confidence interval) of 65% (49–78%) and specificity of 76% (57–89%). Only 32% (12/37) of patients fulfilled both of the main LLC (i.e., non-ischemic myocardial injury/edema with elevated T1 values or presence of late gadolinium enhancement and myocardial edema detected by increased T2 values or positive T2-STIR), resulting in a sensitivity of 32% (20–49%) and specificity of 100% (87–100%). Among controls, 24% (6/25) of patients had elevated native T1 values, but all had normal T2.

**Conclusion:**

In patients with suspected myocarditis due to autoimmune rheumatic disease, who are receiving immunosuppressive therapy, the LLC have a high specificity, but a lower sensitivity than in patients with viral myocarditis. Additional tests should therefore be used to improve disease detection in this population. Where the pre-test probability is high, in patients with suspected myocarditis due to autoimmune rheumatic disease who are undergoing immunosuppression, there may need to be greater reliance on one T1-based criterion rather than both LLC, with the recognition that there is an appreciable rate of raised T1 in controls without myocarditis.

## Graphical abstract

**Figure:**
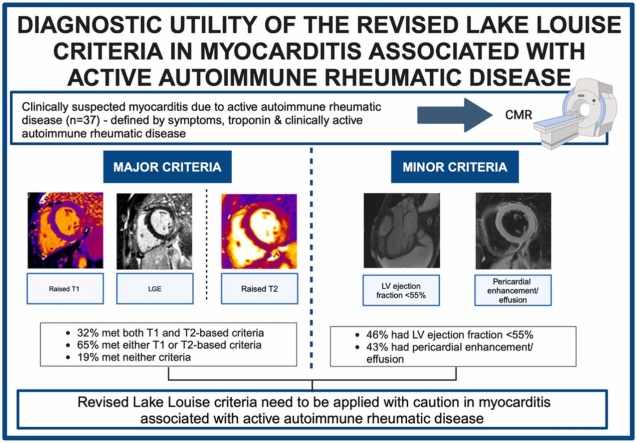
Created with BioRender.com.

## 1. Introduction

The diagnosis of myocarditis due to systemic autoimmune rheumatic disease (AIRD) is challenging due to its atypical or silent clinical presentation [1], [2]. AIRD represents a group of distinct disorders characterized by an overactive immune system resulting in tissue injury and cell death [1]. Myocardial inflammation due to an underlying AIRD frequently mandates an escalation of immunosuppression [3]. While endomyocardial biopsy is regarded as the gold standard for diagnosing myocarditis, advanced cardiac imaging and, in particular, cardiovascular magnetic resonance (CMR) is increasingly supplanting endomyocardial biopsy [4]. Endomyocardial biopsy relies on the histological Dallas criteria, immunohistochemistry, and virus detection to make a diagnosis, however, its application is limited by its invasive nature, high sampling error, and the lack of availability of the expertise required to perform and interpret the test [5].

At present, the CMR diagnosis of myocarditis relies on the revised Lake Louise criteria (LLC), which requires the presence of at least one T1-based and one T2-based marker, ideally incorporating parametric mapping in patients with a high clinical pre-test probability [6], [7]. Although the updated LLC have been validated in large cohorts of patients with viral myocarditis [8], their utility for diagnosing myocarditis due to systemic autoimmune disease is unknown.

While viral myocarditis frequently affects young, often healthy males, myocarditis secondary to AIRD tends to impact older females and is often accompanied by comorbidities such as chronic kidney disease, hypertension and diabetes, which can also increase native T1 values [9], [10]. Elevated native T1 is often seen in several autoimmune conditions, even when the disease is inactive [11]. Moreover, background immunosuppressive treatment may potentially prevent the full expression of myocardial inflammation which also has qualitatively different mechanisms, thereby potentially attenuating the sensitivity of CMR [12]. The diagnostic performance of CMR and the LLC in patients with suspected myocarditis due to AIRD therefore cannot be assumed to be equivalent to that for viral/idiopathic myocarditis, although this assumption is often implicit in clinical practice. There are limited studies that have investigated the utility of the revised LLC in myocarditis due to AIRD, but these studies have only investigated patients with low pre-test probability [13], [14]. We therefore sought to evaluate the diagnostic performance of CMR and the revised LLC in patients with suspected myocarditis due to an underlying AIRD.

## 2. Methods

### 2.1. Study design

Patients with clinically suspected myocarditis due to an underlying active AIRD referred for a CMR scan (Guy’s and St. Thomas’ Hospital, London, UK) from 2020–2024 aged ≥18 years were prospectively recruited. Myocarditis due to AIRD was defined in accordance to the European Society of Cardiology (ESC) diagnostic criteria for suspected myocarditis [15] and refined to include 1) presence of symptoms including chest pain, breathlessness, palpitations, syncope, and/or cardiogenic shock; 2) associated elevated cardiac troponin levels; and 3) clinically active autoimmune disease evidenced by clinical features, physical examination, and serological markers. Exclusion criteria included patients with contraindications to CMR (e.g., intracardiac devices), as well as those who declined consent to study participation. The diagnosis of an autoimmune condition was made by their respective rheumatologists according to the European League Against Rheumatism (EULAR)/American College of Rheumatology (ACR) classification criteria for the respective diseases [16], [17], [18], [19], and the clinical presentation was reviewed by a consensus panel made up of two Professors of Cardiology and a Professor of Rheumatology, blinded to CMR data. Twenty-five patients with an existing autoimmune condition but no suspicion of myocarditis who were referred by their clinicians for routine baseline CMR characterization served as controls. For the control cohort, any patients with a previous diagnosis of myocarditis were excluded. All subjects provided written informed consent. The study complies with the Declaration of Helsinki and was approved by the local institutional review board and the UK National Research Ethics Service (IRAS number 258879/REC reference 20/LO/1076).

### 2.2. Imaging protocol

Studies were performed according to the Society for Cardiovascular Magnetic Resonance (SCMR) guidelines on a 1.5T platform (Siemens Healthineers, MAGNETOM Aera, Forchheim, Germany) [20]. Cine images were acquired in the 2-chamber, 3-chamber, 4-chamber, and a contiguous stack of short-axis views using balanced steady-state free-precession (bSSFP) sequences with the following imaging parameters: flip angle=53˚, 1.9×1.9 mm^2^ in-plane resolution, 8.0 mm slice thickness and bandwidth = 930 Hz/px. A modified Look-Locker inversion recovery sequence (MOLLI) with a 5(3)3 scheme and bSSFP readout was used for T1 mapping with the following parameters: flip angle =50˚, 1.7×1.7 mm^2^ in-plane resolution, 8.0 mm slice thickness, TE/TR = 1.05/2.54 ms and bandwidth=1085 Hz/px. T2-weighted imaging was performed using short tau inversion recovery (STIR) sequence which has a triple-inversion recovery pre-preparation pulses with the following parameters: flip angle = 180˚, 1.4×1.4 mm^2^ in-plane resolution, 8.0 mm slice thickness, TE/TR: 55/576 ms and bandwidth = 849 Hz/px. T2 mapping was performed with a T2-prepared bSSFP sequence with multiple T2 preparation times [0, 28, 55] ms, and 3 recovery heartbeats [21], [22] with the following imaging parameters: flip angle=70˚, 1.9×1.9 mm^2^ in-plane resolution, 8.0 mm slice thickness, TE/TR = 1.06/2.49 ms and bandwidth=1184 Hz/px. Late gadolinium enhancement (LGE) images were acquired using a T1-weighted phase-sensitive inversion recovery (PSIR) sequence with bSSFP readout after administration of 0.15 mmol/kg intravenous gadolinium contrast (Gadobutrol, Bayer Healthcare, Berlin, Germany) [23] with the following imaging parameters: flip angle=50˚, 1.4×1.4 mm^2^ in-plane resolution, 8.0 mm slice thickness, TE/TR = 1.18/2.85 ms and bandwidth = 1085 Hz/px. All tissue characterization, including parametric mapping was performed with whole-heart coverage.

### 2.3. Image analysis

Left ventricular (LV) volumes and ejection fraction were calculated using CVI42 (Circle Cardiovascular Imaging, Version 5.14.2, Calgary, Canada). Matching short-axis slices were compared using cine images, T1 map, T2 map and LGE imaging to ensure analysis of identical slices. For the disease cohort, the region of interest (ROI) was drawn in the areas of abnormal-appearing myocardium at visual evaluation, which signified T2 values above the normal range which has been previously defined in healthy volunteers as per SCMR guidelines [24]. Only myocardial regions with a contiguous area of 40 mm^2^ above the specific thresholds were considered abnormal to reduce the detection of noise as a positive finding [25]. For the control cohort, a single ROI was placed within the septal wall of the mid-ventricular short-axis slice.

### 2.4. Statistical analysis

Statistical analysis was performed using SPSS version 28.11 (IBM Corp., Armonk, New York). Categorical variables are expressed as frequencies and percentages. Continuous variables are reported as medians and interquartile ranges (IQR) or mean ± standard deviation, as appropriate. Comparisons between two groups were made using Fisher's exact test for categorical variables or by the Mann–Whitney U test for continuous variables. Two-tailed values of p<0.05 were considered significant. The overall clinical presentation was regarded as the reference standard for this study. The data that support the findings of this study are available from the corresponding author upon reasonable request.

## 3. Results

### 3.1. Patient characteristics

Thirty-nine patients met our inclusion criteria: one patient declined consent; and one patient died from multiorgan failure before reaching a point of being safe to be scanned. As a result, 37 consecutive patients with clinically suspected myocarditis due to active AIRD were recruited to our study. The median (interquartile range [IQR]) peak troponin and N-terminal pro B-type natriuretic peptide (NT-pro-BNP) levels were 127 (72–318) ng/L (normal range <35 ng/L) and 1022 (276–5120) ng/L (normal range <400 ng/L), respectively. Electrocardiogram (ECG) changes were noted in 28/37 (76%) patients. The commonest symptoms were chest pain (23/37, 62%) and breathlessness (23/37, 62%). The commonest autoimmune condition was systemic lupus erythematosus (SLE) (16/37, 43%), followed by dermatomyositis (6/37, 16%), and eosinophilic granulomatosis with polyangiitis (EGPA) (6/37, 16%).

In keeping with the clinical presentation, the suspected myocarditis group were significantly more symptomatic than the control group (Table 1). Out of the 37 myocarditis patients recruited, 28 patients had troponin T, and 9 patients had troponin I measured, but all were elevated above assay-specific reference ranges. All patients in the control group had normal cardiac biomarkers with a median (IQR) troponin level of 9 (7.5–13.5) ng/L and NT-pro-BNP level of 156 (94–480) ng/L. Among our myocarditis cohort, 11% (4/37) of patients were smokers, 14% (5/37) had hypertension, and 68% (25/37) were on long-term glucocorticoids. There were no differences in cardiovascular risk factors (hypertension, diabetes, hypercholesterolemia, known ischemic heart disease and smoking status) between the myocarditis and control group. For medications at time of CMR, glucocorticoids were the mainstay of treatment in 25/37 (68%) patients, followed by hydroxychloroquine (10/37 [27%]), and intravenous immunoglobulins (9/37 [24%]). Other treatments included cyclophosphamide (8/37 [22%]), rituximab (5/37 [14%]), mycophenolate mofetil (4/37 [11%]), benralizumab (3/37 [8%]), anakinra (2/37 [5%]), and methotrexate (2/37 [5%]).

**Table 1:** Baseline demographic and clinical characteristics.

	Myocarditis (n= 37)	Control (n=25)	p-value
Age, years	43 [22–51]	53 [38–61]	**0.039**
Female, n (%)	29 (78)	18 (72)	0.402
Heart rate, bpm	83±19	77±14	0.285
Body mass index, kg/m^2^	23.6 [20.6–28.7]	23.9 [21.3–28.0]	0.747
Time to CMR, days	6 [2–9]	N/A	N/A
*Clinical characteristics*			
Chest pain, n (%)	23 (62)	8 (32)	**0.020**
Breathlessness, n (%)	23 (62)	6 (24)	**0.003**
Palpitations, n (%)	15 (41)	0 (0)	**<0.001**
Syncope, n (%)	0 (0)	1 (4)	0.403
Cardiogenic shock, n (%)	6 (16)	0 (0)	0.073
ECG changes, n (%)	28 (76)	1 (4)	**<0.001**
Peak high-sensitivity troponin, ng/L	121 [72–318]	9 [7.5- 13.5]	**<0.001**
NT-pro-BNP, ng/L	1022 [276–5120]	156 [94–480]	**0.012**
*Autoimmune diagnosis*			N/A
Systemic Lupus Erythematosus	16 (43)	10 (40)	
EGPA	6 (16)	5 (20)	
Dermatomyositis	6 (16)	1 (4)	
Behçet's disease	3 (8)	0 (0)	
Mixed Connective Tissue Disease	2 (5)	2 (8)	
Leukocytoclastic vasculitis	1 (3)	0 (0)	
Overlap syndrome	1 (3)	0 (0)	
Sjogren's syndrome	1 (3)	1 (4)	
Undifferentiated CTD	1 (3)	0 (0)	
Takayasu’s arteritis	0 (0)	3 (12)	
Granulomatosis with polyangiitis	0 (0)	2 (8)	
Systemic sclerosis	0 (0)	1 (4)	

Values in bold are statistically significant.

*CMR* cardiovascular magnetic resonance, *ECG* electrocardiogram, *NT-pro-BNP* N-terminal pro B-type natriuretic peptide, *EGPA* eosinophilic granulomatosis with polyangiitis, *CTD* connective tissue disease

Data are numbers (%) of cases, means ± standard deviation, or medians [interquartile range].

### 3.2. CMR findings

**Fig. 1:**
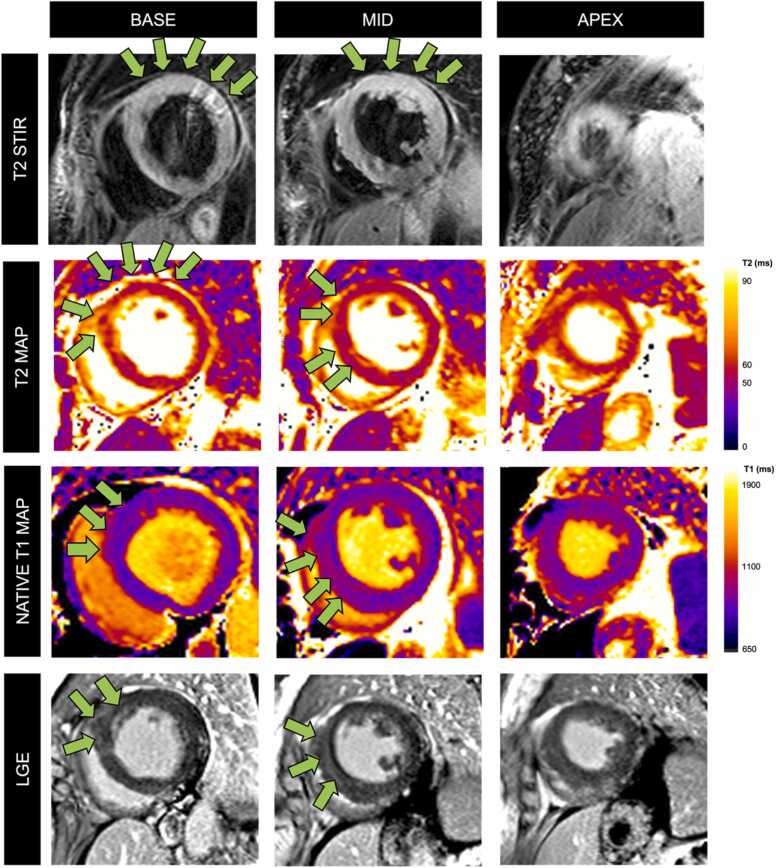
Myocarditis due to active systemic lupus erythematosus (SLE). A 62-year-old female with systemic lupus erythematosus presented with chest pain and breathlessness, associated with raised troponin I level of 177 ng/L (normal range <35 ng/L) and decreased C4 levels of <0.08 g/L (normal range 0.15–0.57 g/L) and raised ESR levels of 97 mm/h (normal range 0–12 mm/h). Transthoracic echocardiography showed moderately impaired LV systolic function with a LV EF of 43%. She was initiated on IV methylprednisolone and immunoglobulin due to highly suspected autoimmune myocarditis secondary to SLE. She subsequently underwent CMR which confirmed lupus myocarditis. T2W-STIR sequences revealed increased myocardial signal intensity. Parametric mapping reveals patchy increased areas of T2 values of 55–59ms (normal range <55 ms) and native T1 values of 1100 ms (normal range 890–1035 ms). LGE images show diffuse mid-wall enhancement. Myocardial injury is depicted by green arrows. C4= complement component 4; *CMR* cardiovascular magnetic resonance, *EF* ejection fraction, *ESR* erythrocyte sedimentation rate, IV intravenous, *LGE* late gadolinium enhancement, *LV* left ventricular, *T2-STIR* T2-short tau Inversion Recovery

The CMR findings are summarized in Table 2. The median LV ejection fraction (EF) (IQR) was lower in the diseased cohort than the controls (58% [45–61%] *versus* 61% [57–66%] in the controls, p=0.006). The right ventricular (RV) EF (IQR) in the diseased cohort was also significantly lower (Table 2). An LGE pattern typical of an autoimmune myocarditis/vasculitis was observed in 14/37 (38%) patients in the active disease cohort. Five patients had epicardial to mid-myocardial enhancement in the inferolateral, inferior or lateral segments, 4 patients had diffuse mid-wall enhancement in the mid-septum, 4 patients had widespread diffuse enhancement in all myocardial segments, and 1 patient had subendocardial enhancement typical of acute cardiac EGPA [4]. An example of a patient with acute lupus myocarditis is shown in Fig. 1. No LGE was noted in the control group.

**Table 2:** Cardiovascular magnetic resonance findings.

	Diseased (n=37)	Control (n=25)	p-value
*Overall CMR findings*			
LV EF, %	58 [45–61]	61 [57–66]	**0.006**
RV EF, %	54 [46–58]	59 [55–62]	**0.002**
Indexed LV EDV, mL/m^2^	84 [74–97]	78 [72–90]	0.438
Indexed RV EDV, mL/m^2^	86 [72–102]	83 [78–93]	0.859
*Late Gadolinium Enhancement, n (%)*	14 (38%)	0 (0)	**<0.001**
Epicardial to mid-inferolateral/ inferior/ lateral	5		
Mid-wall enhancement of the septum	4		
Diffuse enhancement of all segments	4		
Multifocal subendocardial enhancement (vasculitic pattern)	1		
*Main Lake Louise criteria, n (%)*			
T1-based criteria abnormality/Non-ischemic myocardial injury (Abnormal T1/LGE)	23 (62)	6 (24)	**0.003**
T2-based criteria abnormality/ Myocardial edema (Abnormal T2/positive STIR)	13 (35)	0 (0)	**<0.001**
Both main criteria (abnormal T1/LGE *and* abnormal T2/positive T2-STIR)	12 (32)	0 (0)	**<0.001**
Either main criterion (abnormal T1/LGE *or* abnormal T2/positive T2-STIR)	24 (65)	6 (24)	**0.002**
*Supportive Lake Louise criteria, n (%)*			
Pericardial effusion or increased signal on T2-STIR	16 (43)	0 (0)	**<0.001**
LV dysfunction (EF <55%)	17 (46)	0 (0)	**<0.001**
*Criteria, n (%)*			
Elevated T1 values	21 (57)	6 (24)	**0.011**
Elevated T2 values	13 (35)	0 (0)	**<0.001**
No evidence of ischemic injury or myocardial edema defined by elevated T1 or T2 values or a positive STIR	12 (32)	19 (76)	**0.003**
No evidence of ischemic injury or myocardial edema defined by elevated T1 or T2 values or a positive STIR *or* minor criteria (presence of LV function <55% or pericardial enhancement/ effusion) met	7 (19)	19 (76)	**<0.001**

Values in bold are statistically significant.

*LV* left ventricular, *RV* right ventricular, *EDV* end diastolic volume, *LGE* late gadolinium enhancement, *STIR* short tau inversion recovery, *EF* ejection fraction

Data are numbers (%) of cases or medians [interquartile range]”.

### 3.3. Lake Louise criteria and myocarditis due to active AIRD

Only twelve patients (12/37, 32%) diagnosed with acute autoimmune myocarditis fulfilled two of the main LLC, comprising one T1-based abnormality defined by either abnormal T1 values or the presence of LGE, and one T2-based abnormality as defined by abnormal T2 values and/or positive T2-STIR. Twenty-four patients (24/37, 65%) met either of the main criteria, i.e., a T1-based or a T2-based abnormality. Twenty-three patients (23/37, 62%) met a T1-based criterion (either elevated native T1 values and/or LGE presence), and among the whole cohort, thirteen patients (13/37, 35%) had a T2-based abnormality. In terms of mapping, 21 patients (21/37, 57%) had abnormal T1 values, and 13 patients (13/37, 35%) had abnormal T2 values.

The mean T1 (±standard deviation) in patients who fulfilled the non-ischemic myocardial injury criterion in our diseased cohort was significantly higher at 1113 (±60) ms compared to patients who did not fulfill the criterion at 984 (±29) ms, p<0.001 (scanner-specific normal range: 890–1035 ms). The mean T2 (±standard deviation) in patients who fulfilled the myocardial edema criterion was significantly higher at 60 (±3) ms compared to patients who did not fulfill the criterion at 47 (±3) ms, p<0.001 (scanner-specific normal range: <55 ms).

Among the clinically suspected myocarditis group, 16/37 (43%) patients had a pericardial effusion or inflammation, and 17/37 (46%) patients had a reduction of LVEF <55%, whereas none of the controls exhibited these features. Despite having a high pre-test probability of autoimmune myocarditis, seven (7/37, 19%) patients had an ostensibly normal CMR scan (i.e., no evidence of major criteria defined by normal T1 and T2 values, normal T2-STIR images and no LGE and minor criteria defined by no evidence of pericardial effusion or inflammation and LV EF >55%). In our control cohort, six (6/25, 24%) patients had elevated native T1 values, but none demonstrated raised T2.

The diagnostic performance of CMR is summarized in the confusion matrix in Table 3. The overall sensitivity (95% confidence interval [CI]) of the combined revised LLC in diagnosing myocarditis due to an active AIRD was 32% (20–49%) with a specificity of 100% (87–100%), positive predictive value (PPV) of 100% (76–100%), and negative predictive value (NPV) of 50% (37–64%). The sensitivity of either of the two revised LLC was 65% (49–78%) with a specificity of 76% (57–89%), a PPV of 80% (63–90%), and NPV of 59% (42–74%). The sensitivity of T1 mapping in diagnosing myocarditis secondary to active AIRD was 57% (41–71%) with a specificity of 76% (56–89%), PPV of 78% (59–89%), and NPV of 54% (39–70%). The sensitivity of T2 mapping in diagnosing myocarditis associated with active AIRD was 35% (22–51%) with a specificity of 100% (87–100%), PPV of 100% (77–100%), and NPV of 51% (37–64%). The results were similar when examining a sub-group of patients with just SLE, which was the most common underlying autoimmune disease in the cohort (Supplementary Table 1).

**Table 3:** Confusion matrix summarizing the diagnostic performance of CMR.

	Diagnosis positive	Diagnosis negative
*Two LLC fulfilled*	12	0
*Two LLC not fulfilled*	25	25
Sensitivity (95% CI): 32% (20–49%), specificity: 100% (87–100%); positive predictive value: 100% (76–100%); negative predictive value: 50% (37–64%); positive likelihood ratio: undefined; negative likelihood ratio: 0.68 (0.54- 0.84)		
*One LLC fulfilled*	24	6
*One LLC not fulfilled*	13	19
Sensitivity (95% CI): 65% (49–78%), specificity: 76% (57–89%); positive predictive value: 80% (63–90%); negative predictive value: 59% (42–74%); positive likelihood ratio: 2.70 (1.29- 5.65); negative likelihood ratio: 0.46 (0.28–0.75)		
*Elevated T1 values - positive*	21	6
*Normal T1 values - negative*	16	19
Sensitivity (95% CI): 57% (41–71%), specificity: 76% (56–89%); positive predictive value: 78% (59–89%); negative predictive value: 54% (39–70%); positive likelihood ratio: 2.36 (1.11–5.02); negative likelihood ratio: 0.57 (0.37–0.87)		
*Elevated T2 values - positive*	13	0
*Normal T2 values - negative*	24	25
Sensitivity (95% CI): 35% (22–51%), specificity: 100% (87–100%); positive predictive value: 100% (77–100%); negative predictive value: 51% (37–64%); positive likelihood ratio: undefined; negative likelihood ratio: 0.65 (0.51–0.82)		

*CI* confidence interval, *LLC* Lake Louise criteria

2 LLC fulfilled was defined as both main criteria (abnormal T1/LGE *and* abnormal T2/positive T2-STIR)

1 LLC fulfilled was defined as either main criterion (abnormal T1/LGE *or* abnormal T2/positive T2-STIR)

Data are numbers of cases.

### 3.4. Characteristics of LLC positive cohort

The overall median (IQR) time for the entire cohort from peak troponin levels and presence of symptoms to the CMR scan was 6 (2–9) days (Table 4). CMR scans were performed as soon as it was deemed clinically safe as 16% of the patients in our cohort had cardiogenic shock and were critically ill, thereby delaying their CMR examinations. Nevertheless, the median (IQR) time to scan of patients who fulfilled the LLC was 7 (3–11) days. In a sensitivity analysis, this was not statistically different from patients that did not fulfill the main LLC with a median (IQR) time to CMR scan of 6 (2–9 days), p=0.259 (Table 4). The peak troponin levels were significantly higher in the LLC positive group with median (IQR) levels of 191 (120–920) ng/L compared to the LLC negative group with median (IQR) levels of 83 (58–229) ng/L, p=0.022. The peak NT-pro BNP levels and medications did not differ significantly between the LLC positive and LLC negative group (Table 4). There was also no significant difference in the rates of treatment intensification pre-scan between those with positive and negative CMR findings (Table 4).

**Table 4:** Characteristics of patients stratified by fulfillment of the revised Lake Louise Criteria.

	LLC positive (n=12)	LLC negative (n=25)	p-values
Time to scan, days	6 (2–9)	7 (3–11)	0.259
Peak troponin, ng/L	191 (120–920)	83 (58–229)	**0.022**
Peak NT-pro BNP, ng/L	1490 (436–6969)	950 (181–5149)	0.635
eGFR, mL/min/1.73 m^2^	90 (66–92)	90 (71–113)	0.542
*Medications, n (%)*			
Glucocorticoids	7 (58)	18 (72)	0.468
Hydroxychloroquine	3 (25)	7 (28)	1.000
Intravenous immunoglobulin	3 (25)	6 (24)	1.000
Cyclophosphamide	3 (25)	5 (20)	1.000
Rituximab	1 (8)	4 (16)	1.000
Mycophenolate	2 (17)	2 (8)	0.582
Anakinra	0 (0)	2 (8)	1.000
Methotrexate	0 (0)	1 (4)	1.000
Benralizumab	0 (0)	3 (12)	0.537
Treatment intensification	9 (75)	17 (68)	0.737

Values in bold are statistically significant.

*LLC* Lake Louise criteria, *NT-pro BNP* N-terminal pro B-type natriuretic peptide, *eGFR* estimated glomerular filtration rate

Data are numbers (%) of cases or medians [interquartile range]”.

## 4. Discussion

To the best of our knowledge, this is the first study that has comprehensively evaluated the utility of the revised LLC in patients with clinically suspected acute myocarditis associated with active AIRD using contemporary multiparametric CMR. The latter was relatively insensitive and only 12/37 (32%) patients fulfilled the full revised LLC. There was a relatively high prevalence of abnormalities in native T1 (21/37, 57%), however, a substantial proportion of the controls also exhibited this (6/25, 24%), lowering its specificity.

Myocarditis secondary to active AIRD is a rare and challenging diagnosis, and to the best of our knowledge, our study represents the largest cohort of patients with suspected myocarditis due to a spectrum of systemic autoimmune diseases studied with multiparametric CMR thus far. Only two studies have investigated the utility of both the original and revised LLC in diagnosing cardiac involvement in AIRD [13], [14]. Markousis-Mavrogenis *et al*. showed that 76% of systemic sclerosis patients with suspicion of myocardial inflammation fulfilled the revised LLC [13]. However, it is unclear what criteria were used to define "suspicion of myocardial inflammation" as no data regarding clinical characteristics, biomarkers, and ECG changes were presented [13]. Meloni *et al.* also studied patients with systemic sclerosis and found that only 59.6% of patients fulfilled the LLC, however their patient group had a low probability of myocardial inflammation with a mean troponin of 3.73 ng/L (7.43) [14].

Our current study demonstrated that the sensitivity of the full LLC in myocarditis associated with active AIRD was low at 32% and was limited by the detection of myocardial edema. Patients that met both LLC criteria in our cohort had significantly higher peak troponin levels than those that did not fulfill the LLC (191 *vs* 83 ng/L, p=0.029). In contrast, Luetkens *et al.* found that the revised LLC perform well in idiopathic/viral myocarditis with an overall sensitivity of 87.5% and specificity of 96.2%, despite a mean troponin I level of 13.9 ng/L [8]. The performance of the LLC improved when only one criterion was used given that the disease cohort had a high pre-test probability of myocarditis. However, the performance was still lower than typically seen in patients with viral myocarditis, where the specificity of elevated native T1 is likely to be far higher [6].

In viral/idiopathic myocarditis, patients are typically young, and often male, with no other comorbidities and frequently, the heart is the only organ involved in their disease [7]. In contrast, myocarditis due to AIRD tends to affect older females often with a background of long-term glucocorticoid use and pre-existing cardiovascular comorbidities such as chronic kidney disease, hypertension, and diabetes [26]. In our control cohort, we observed that almost a quarter of patients had elevated native T1 values. Thus, in contradistinction to the situation with viral myocarditis, isolated abnormalities in native T1 cannot be relied upon as a marker of myocardial edema. Other studies have also found that among stable patients with autoimmune conditions, native T1 values are often elevated [11].

In their study of patients with suspected immune-checkpoint inhibitor myocarditis, Thavendiranathan *et al*. demonstrated abnormal native T1 and T2 values in 78% and 43% respectively [27]. However, echoing our findings, only 48% of the patients met both main LLC. This illustrates that the revised LLC, which were validated in patients with a viral/idiopathic myocarditis, need to be applied with caution to other etiologies of clinically suspected myocarditis. Thus, CMR cannot be assumed to have the same diagnostic performance in all clinical settings.

In our cohort of patients with high pre-test probability, normal CMR scans were observed in 7/37 (19%) patients, despite a comprehensive CMR examination with whole-heart coverage with T1 and T2 mapping, STIR, and LGE imaging. It remains challenging to account for their clinical presentation as these patients had significant troponin elevation without an alternative plausible cause. There are several plausible reasons for this. First, it is possible that the presence of inflammation in these patients is simply below the sensitivity of CMR to detect edema. Our T2 mapping sequence employs a 2.0 × 2.0 × 8 mm^3^ resolution and the presence of inflammation below this resolution may have been missed. Second, this may relate to the timing of CMR. It is widely accepted that the optimal time to detect myocardial edema on CMR from clinical presentation is up to 14 days [28]. However, the median time in our cohort of patients with normal scan was just 7 days. A sensitivity analysis also showed no significant difference in timing between patients who did and did not fulfill the revised LLC. Third, it remains unknown if immunosuppression therapy had an overall impact on resultant CMR findings. Among the seven patients with ostensibly normal scans, all but one patient was on some form of immunosuppression. However, all patients, and even the controls had received some form of immunosuppression, and there were no significant differences in the rates of treatment intensification prior to the scans between those with positive and negative CMR findings.

## 5. Limitations

First, endomyocardial biopsy, which is regarded as gold standard to diagnose acute myocarditis, was not performed in our cohort. However, this is now rarely performed in clinical practice due to its invasive nature, risk of sampling error, and lack of expertise in performing and interpreting biopsies [7]. We therefore chose a pragmatic and practical approach to make a diagnosis of clinically suspected myocarditis based on clinical features, biomarkers, and ECG changes. This mirrors clinical practice. Additionally, the standardized approach of applying the ESC criteria as a reference standard is widely used in CMR-related myocarditis studies [25]. We applied additional criteria above what is required according to the ESC criteria when determining the inclusion criteria for this study, mandating a troponin elevation, and evidence of clinically active AIRD, as well as validation from an independent panel. Secondly, we were not able to investigate the potential effects of immunosuppression on T1 and T2 values, although there were no differences seen in our LLC positive and negative cohort. Given the potentially life-threatening implications of cardiac involvement in patients with systemic AIRD, patients are often initiated on immunosuppression while undergoing further investigations. It is therefore possible that CMR findings may have been attenuated by early treatment. However, this reflects real-world clinical practice, and the performance of the revised LLC needs to be interpreted through this lens given that in contrast, for patients with viral/idiopathic myocarditis, care is supportive and not likely to abrogate CMR findings. Thirdly, in our control cohort, approximately a third (32%) of the patients had non-specific chest pain. However, the presence of symptoms is challenging to interpret in these complex diseases, where there is often multi-system involvement. Furthermore, these patients did not have clinically active autoimmune disease, and the median troponin level was only 9 ng/L, and within reference ranges, thereby excluding the diagnosis of myocarditis. None of the controls exhibited any elevation of myocardial T2 or any supportive features of myocarditis on imaging or clinically. Fourthly, cardiac troponin T can be expressed in skeletal muscle following injury [29]. However, of the 37 patients studied, only 6 had any history of skeletal muscle involvement in their autoimmune rheumatic disease (dermatomyositis) and of these, only 4 had troponin T rather than troponin I assayed. Furthermore, to reduce the risk of false positives, all patients studied had to have clinical symptoms and/or other supportive features in the setting of a clinically active autoimmune disease to be included.

Our overall sample size remains small; however, this is a rare pathology. Furthermore, our institution is a quaternary referral center for complex autoimmune diseases with a dedicated cardio-rheumatology service and access to 6 dedicated CMR scanners. This is likely to be atypical compared to most centers, yet despite this, and prompt access to advanced imaging, the number of patients meeting CMR diagnostic criteria for myocarditis was lower than that typically seen in patients with suspected viral/idiopathic myocarditis. It is likely that in centers without direct access to CMR or limited internal access, the sensitivity of the technique may be even further attenuated. Finally, while our cohort was ethnically very diverse, reflecting the population of south London and southeastern England that we serve, men were relatively underrepresented making up only 8 out of 37 patients (22%) in the myocarditis arm. However, this is likely representative of the epidemiology of autoimmune rheumatic disease, which is more prevalent among women rather than any latent bias in recruitment [30].

## 6. Conclusions

In patients with suspected myocarditis due to autoimmune rheumatic disease, who are receiving immunosuppressive therapy, the Lake Louise Criteria have a high specificity, but a lower sensitivity than in patients with viral myocarditis. Additional tests should therefore be used to improve disease detection in this population. Diagnostic performance equivalent to that seen in viral/idiopathic myocarditis, where a high proportion of patients meet both criteria, cannot be assumed, and the specificity of raised T1 is likely to be impaired due to the high prevalence of comorbidities also associated with T1 abnormalities. While specific, elevated T2 had a low sensitivity for myocarditis in patients with autoimmune disease. Nevertheless, where the pre-test probability is high, in patients with suspected myocarditis due to autoimmune rheumatic disease who are receiving immunosuppression, there may need to be greater reliance on one T1-based criterion rather than both LLC, with the recognition that there is an appreciable rate of raised T1 in controls without myocarditis. Further work is required to define the role and limitations of CMR for the diagnosis of myocarditis in patients with systemic immune-mediated inflammatory diseases, and a diverse range of other etiologies of inflammatory cardiomyopathy.

## Funding

This work was supported by British Heart Foundation CRTF FS/20/13/34857 (fellowship grant for AH) and by the Department of Health through the National Institute for Health Research (NIHR) comprehensive Biomedical Research Centre award to Guy’s & St Thomas’ NHS Foundation Trust in partnership with King’s College London and King’s College Hospital NHS Foundation Trust and by the NIHR MedTech Co-operative for Cardiovascular Disease at Guy’s and St Thomas’ NHS Foundation Trust. No funder had any role in study design, data collection, analysis, or any influence on the content of the final manuscript.

## Author contributions

A.H. and T.F.I. were involved in the conception and design of this study. A.H., G.S., B.L., D.D.C. and M.F. were involved with patient recruitment. R.R., M.M. and D.D.C. adjudicated all patients. A.H., B.D. and T.F.I. were involved with data acquisition, interpretation, and analysis of diseased and control cohorts. A.H. and T.F.I. were involved with statistical analysis and generating the first draft of this manuscript. All authors participated in the revision and final approval of this manuscript.

## Ethics approval and consent

This study was approved by the National Research Ethics Service (REC 15/NS/0030). Written informed consent was obtained for all subjects and healthy volunteers. Anonymized data were analyzed at the School of Biomedical Engineering and Imaging Sciences (King’s College London) at St. Thomas’ Hospital.

## Consent for publication

All subjects provided written informed consent for the publication of anonymized images in this manuscript. The consent forms are held in the patients’ clinical notes and are available to the Editor-in-Chief upon request.

## Declaration of competing interests

The authors declare the following financial interests/personal relationships which may be considered as potential competing interests: Alina Hua reports financial support and travel were provided by British Heart Foundation. NA reports administrative support was provided by NIHR Biomedical Research Centre at Guy’s and St Thomas’ NHS Foundation Trust and King’s College London. If there are other authors, they declare that they have no known competing financial interests or personal relationships that could have appeared to influence the work reported in this paper.

## Data Availability

Data generated or analyzed during the study are available from the corresponding author by request.
