# Supplementary material for: Diagnostic utility of the revised Lake Louise criteria in myocarditis associated with active autoimmune rheumatic disease
Source: J Cardiovasc Magn Reson. 2025 Jun 2;27(2):101916. doi: 10.1016/j.jocmr.2025.101916 (PMC12445391; doi:10.1016/j.jocmr.2025.101916)
Supplement: Supplementary file 1 — Supplementary material [file mmc1.docx]

**Supplementary Table 1. Cardiovascular Magnetic Resonance findings in the sub-group of patients with Systemic Lupus Erythematosus**

|  | **SLE (n=16)** | **Control (n=10)** | **p-value** |
| --- | --- | --- | --- |
| **Overall CMR findings** |  |  |  |
| LV EF, % | 47 [39-58] | 65 [60-68] | **<0.001** |
| RV EF, % | 52 [48-59] | 60 [55-65] | **0.025** |
| Indexed LV EDV, ml/m^2^ | 90 [74-104] | 76 [72-86] | 0.234 |
| Indexed RV EDV, ml/m^2^ | 84 [59-107] | 85 [79-93] | 0.760 |
| **Late Gadolinium Enhancement, n (%)** | 7 (44) | 0 (0) | **0.023** |
| Epicardial to mid-inferolateral/ inferior/ lateral | 2 |  |  |
| Mid-wall enhancement of the septum | 1 |  |  |
| Diffuse enhancement of all segments | 4 |  |  |
| Multifocal subendocardial enhancement (vasculitic pattern) | 0 |  |  |
| **Main Lake Louise Criteria, n (%)** |  |  |  |
| T1-based criteria abnormality/Non-ischemic myocardial injury (Abnormal T1/LGE) | 11 (69) | 3 (30) | **<0.001** |
| T2-based criteria abnormality/ Myocardial edema (Abnormal T2/positive STIR) | 7 (44) | 0 (0) | **0.016** |
| Both main criteria (abnormal T1/LGE *and* abnormal T2/positive T2-STIR) | 6 (38) | 0 (0) | **0.030** |
| Either main criterion (abnormal T1/LGE *or* abnormal T2/positive T2-STIR) | 12 (75) | 3 (30) | **0.025** |
| **Supportive Lake Louise Criteria, n (%)** |  |  |  |
| Pericardial effusion or increased signal on T2-STIR | 9 (56) | 0 (0) | **0.004** |
| LV dysfunction (EF <55%) | 11 (69) | 0 (0) | **<0.001** |
| **Criteria, n (%)** |  |  |  |
| Elevated T1 values | 11 (69) | 3 (30) | 0.060 |
| Elevated T2 values | 7 (44) | 0 (0) | **0.016** |
| No evidence of ischemic injury or myocardial edema defined by elevated T1 or T2 values or a positive STIR | 4 (25) | 7 (70) | 0.087 |
| No evidence of ischemic injury or myocardial edema defined by elevated T1 or T2 values or a positive STIR *or* minor criteria (presence of LV function <55% or pericardial enhancement/ effusion) met | 2 (13) | 7 (70) | **0.009** |

Values in bold are statistically significant.

SLE=Systemic Lupus Erythematosus; LV=left ventricular; RV=right ventricular; EDV=end diastolic volume; LGE= late gadolinium enhancement; STIR= short tau inversion recovery; EF=ejection fraction.

**Diagnostic performance of LLC in the SLE sub-group**

Only six patients (38%) diagnosed with acute SLE myocarditis fulfilled two of the main LLC, comprising one T1-based abnormality defined by either abnormal T1 values or the presence of LGE, and one T2-based abnormality as defined by abnormal T2 values and/or positive T2-STIR. Twelve (75%) met either of the main criteria, *i.e.,* a T1-based or a T2-based abnormality. Eleven (69%) met a T1-based criterion (either elevated native T1 values and/or LGE presence), and among the whole cohort, seven patients (44%) had a T2-based abnormality. In terms of mapping, 11 patients (69%) had abnormal T1 values, and 7 patients (44%) had abnormal T2 values.

The mean T1 (±standard deviation) in patients who fulfilled the non-ischemic myocardial injury criterion in our diseased SLE cohort was significantly higher at 1124 (±66) ms compared to patients who did not fulfil the criterion at 974 (±17) ms, p =0.001 (scanner-specific normal range: 890-1035 ms). The mean T2 (±standard deviation) in patients who fulfilled the myocardial edema criterion was significantly higher at 61 (±4) ms compared to patients who did not fulfil the criterion at 48 (±3) ms, p <0.001 (scanner specific normal range: <55ms).

The overall sensitivity (95% confidence interval [CI]) of the combined revised LLC in diagnosing myocarditis due to active SLE was 38% (18-61%) with a specificity of 100% (72-100%), positive predictive value (PPV) of 100% (61-100%) and negative predictive value (NPV) of 50% (30-70%). The sensitivity of either of the two revised LLC was 75% (51-90%) with a specificity of 70% (40-89%) with a PPV of 80% (55-93%) and NPV of 64% (35-85%). The sensitivity of T1 mapping in diagnosing myocarditis secondary to active SLE was 69% (44-86%) with a specificity of 70% (40-89%), PPV of 79% (52-92%) and NPV of 58% (32-81%). The sensitivity of T2 mapping in diagnosing myocarditis associated with active SLE was 44% (23-67%) with a specificity of 100% (72-100%), PPV of 100% (65- 100%), and NPV of 53% (32-73%).
